# Kidney function decline associated with proton pump inhibitors: results from the ELSA-Brasil cohort

**DOI:** 10.1186/s12882-023-03300-4

**Published:** 2023-09-28

**Authors:** Andrêza Soares dos Santos, Sara Teles de Menezes, Isabella Ribeiro Silva, William Neves Oliveira, Mariana Linhares Pereira, José Geraldo Mill, Sandhi Maria Barreto, Roberta Carvalho Figueiredo

**Affiliations:** 1 Postgraduate Program in Health Sciences, Universidade Federal de São João del-Rei, Sebastião Gonçalves Coelho Street, 400 – Chanadour, Divinópolis, 35501-296 MG Brazil; 2 Longitudinal Study of Adult Health – ELSA-Brasil, Medical School & Clinical Hospital/EBSERH, Universidade Federal de Minas Gerais, Belo Horizonte, MG Brazil; 3 Department of Physiological Sciences & University Hospital, Universidade Federal do Espírito Santo, Vitória, Brazil; 4 Medical School & Clinical Hospital/EBSERH, Universidade Federal de Minas Gerais, Belo Horizonte, MG Brazil

**Keywords:** Proton pump inhibitors, Chronic kidney disease, Kidney function, Pharmacovigilance, Pharmacoepidemiology

## Abstract

**Objective:**

Investigate the longitudinal association of use and time of use of proton pump inhibitors (PPI) with incidence of chronic kidney disease (CKD) and kidney function change.

**Methods:**

Prospective study with 13,909 participants from baseline (2008–2010) and second wave (2012–2014) of the ELSA-Brasil (mean interval between visits = 3.9 years (1.7–6.0)). Participants answered about use and time use of the PPI in the two weeks prior the interview. Renal function was assessed by glomerular filtration rate estimated by the Collaboration Equation for the Epidemiology of Chronic Kidney Disease. Values below 60ml/min/1.73 m² in wave 2 were considered incident CKD. Associations between PPI use and time of use at baseline and incident CKD and decline in renal function were estimated, respectively, by logistic regression and linear models with mixed effects, after adjusting for confounders.

**Results:**

After adjustments, PPI users for more than six months had an increased risk of CKD compared to non-users. Compared to non-users, users PPIs for up to six months and above six months had greater decline in kidney function over time.

**Conclusion:**

This cohort of adults and elderly, after a mean interval of 3.9 years, PPI use and initial duration were associated with kidney function change between visits.

## Introduction

Proton pump inhibitors (PPIs) are drugs used for gastric disorders. Because of their low toxicity, they are overprescribed and not always used rationally [1, 2]. Although PPIs are safe, observational studies suggest that PPI use is associated with an increased risk of several adverse health events [3, 4], including acute kidney injury (AKI) [5], development and progression of chronic kidney disease (CKD) [6–8] and end-stage renal disease (ESRD) [7, 8].

According to the Kidney Disease Improvement Global Outcomes (KDIGO) [9], CKD is characterized by persistent kidney damage (more than three months), usually identified by a glomerular filtration rate (GFR) < 60 mL/min/1.73 m^2^ or urinary albumin creatinine ratio ≥ 30 mg/g. In developed countries, the estimated prevalence of CKD ranges from 5 to 15% [10, 11]. In Latin America, these data are still scarce, although some studies show an increasing number of people on renal replacement therapy [12, 13]. In a large epidemiological study in Brazil, the prevalence of CKD was estimated at 6.6% (95%CI 6.0–7.4%) among adults [14]. The prevalence of CKD was determined in baseline exams (2008–2010) of the Longitudinal Health Study (ELSA-Brasil) and 8.9% had a glomerular filtration rate < 60 mL/min/1.73 m^2^ [15].

The mechanisms supporting the association between PPI use and loss of kidney function resulting in CKD are still unclear. Studies suggest the association between these drugs and acute interstitial nephritis (AIN) [16–19]. About 30–70% of individuals with AIN do not fully recover kidney function [17]. Incomplete recovery of renal function associated with PPI-induced chronic interstitial nephritis can lead to the development of AKI and subsequent CKD [16, 20]. The relationship between AKI and subsequent CKD development is supported by several studies, suggesting an important role in the global CKD epidemiology and ESRD on a bidirectional axis between AKI and CKD [21]. PPI use can also cause severe hypomagnesemia [22, 23], which is associated with a declining GFR rate in individuals with CKD [24], type II diabetes mellitus [25] and incidence of CKD [26].

Although the mechanisms are uncertain, the evidence linking PPI use and negative renal outcomes is consistent and would not be explained by confounding [26]. CKD is a major public health problem, as it is associated with a higher risk of cardiovascular and general mortality, in addition to social and individual costs [27]. Presenting evidence on the association between PPIs and incidence of CKD is important, as well as evaluating the decline in renal function associated with the use of PPIs, provides fundamental information for early treatments. Furthermore, evidence on the PPI use and kidney function association varies according to the duration of medication use is still scarce and controversial [27–29]. Thus, this study aimed to prospectively investigate whether regular PPI use at baseline is associated with change in GFR and incident kidney disease after a follow-up of about four years in a sample of middle-aged and elderly adults in the cohort. ELSA-Brazil.

## Methods

### Study design

A prospective cohort study with baseline (2008–2010) and follow-up (wave 2: 2012–2014) from ELSA-Brasil. The ELSA-Brasil comprises 15,105 active or retired employees, 35–74 years of age at baseline, from universities or research institutions located in six Brazilian capitals (Belo Horizonte, Porto Alegre, Rio de Janeiro, Salvador, Sao Paulo, Vitoria). ELSA-Brasil includes volunteers (76% of the final sample) and actively recruited participants (24%), the latter being recruited from listings of civil servants. Other publications contain detailed information on the ELSA-Brasil design and baseline data [30, 31].

### Study population

**Fig. 1:**
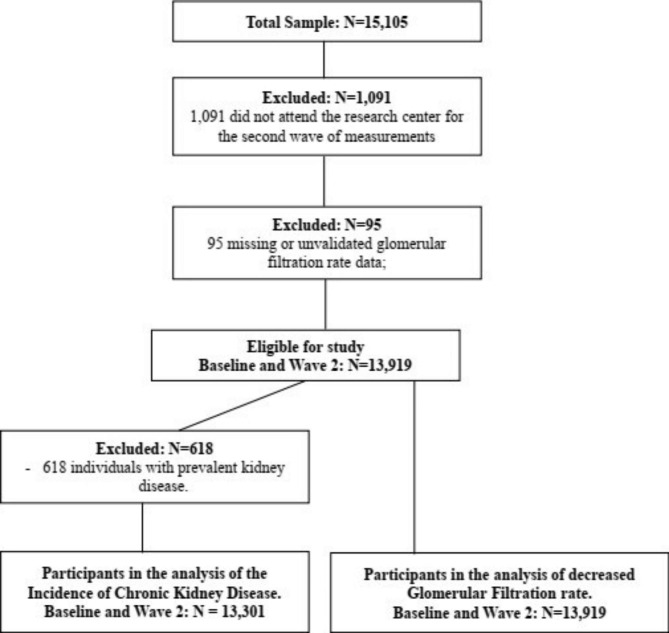
Study population flowchart

Of the 15,105 baseline participants, 1,091 (7.2%) did not attend the research center for the second wave of measurements, of which 223 (20.4%) died. Thus, 14,014 subjects (94% of the eligible population) completed the second wave and 95 subjects with missing GFR data at baseline or wave 2 were excluded. For the analysis of drug use time, 313 PPI users with use time missing data were also excluded in the analysis of change in Glomerular Filtration Rate and 297 PPI users with use time missing data in the analysis of the Incidence of Chronic Kidney Disease. Sample selection is described in Fig. 1.

### Study variables

#### Outcomes

We used the continuous measurements of estimated glomerular filtration rate (eGFR) obtained in the baseline and wave 2, and the incident CKD (defined as GFR < 60 ml/min/1.73 m^2^ in wave 2, according with KDIGO 2012 (Clinical Practice Guideline for the Evaluation and Management of Chronic Kidney Disease) [9]. The GFR was estimated using the Chronic Kidney Disease Epidemiology Collaboration equation (CKD-EPI) [32] without correction for races, as detailed in Barreto et al. [15].

Creatinine was evaluated in serum samples by the kinetic method, according to Jaffé (Advia 1200; Siemens, Munich, Germany), by applying a conversion factor derived from the calibration sample for isotope dilution mass spectrometry, as recommended by the National Kidney Disease Education Program [33].

#### Exposure

All participants were instructed to take medication prescriptions, packages, and inserts used in the last two weeks before the interview and report the time of use of each medication. All medicine trade names were converted either to the Brazilian common denomination (BCD) or international common denomination (INN) and the time of use to months. PPI users were individuals who, at baseline, reported using PPI class drugs (omeprazole, esomeprazole, lansoprazole, pantoprazole, and rabeprazole) regularly in the last two weeks prior to the interview.

According to the guidelines, the duration of PPI use should be short-term (2 to 12 weeks), after which PPI therapy should be discontinued unless maintenance therapy is clearly indicated. Thus, the time of use was stratified into non-users (no PPI use at baseline), users up to six months and above six months [34–36].

#### Covariates

The covariates were obtained through standardized face-to-face interviews and clinical procedures at the ELSA-Brasil baseline [30].

Sociodemographic characteristics included sex and per capita household income (distributed by quintiles). The variable age was used to index time. Thus, the baseline age of each individual was the starting point, and the age at wave 2 corresponded to the baseline age plus the interval in years between the two visits ([date of wave 2 visit - date of baseline visit] / 365.25). Confounding factors were excessive alcohol consumption, defined as intake of ≥ 210 g of ethanol per week for men and ≥ 140 g per week for women [37], smoking was assessed by the following questions: “Are you or have you ever been a smoker, that is, have you smoked at least 100 cigarettes (five packs of cigarettes) throughout your life?” and “Do you currently smoke cigarettes?,” they were classified as never smokers, ex-smokers, and current smokers, obesity (body mass index ≥ 30 kg/m^2^), diabetes and hypertension (self-reported); cardiovascular disease (CVD) (self-reported medical diagnosis of the following comorbidities: acute myocardial infarction, angina, congestive heart failure, stroke or myocardial revascularization) and use of non-steroidal anti-inflammatory drugs (NSAIDs), Angiotensin II receptor blockers (ARBs) and Angiotensin-converting enzyme inhibitors (ACE).

### Statistical analysis

Categorical variables were described as proportions and continuous variables either as medians and interquartile ranges or means and standard deviation (SD).

The association between PPI use and duration of use (both at baseline) with the incidence of CKD was investigated using binary logistic regression with no CKD incident as reference category. Crude and adjusted Odds Ratio (OR) and respective 95% confidence intervals (95%CI), were estimated. First, we estimated the crude OR of the correlation between PPI use and CKD (Model 0), then we adjust to age, sex, and per capita household income (Model 1). Subsequently, Model 1 was adjusted for excessive alcohol consumption, smoking and obesity (Model 2). Finally, CVD, diabetes, and hypertension data were added to model 2 (Model 3). The adjustment for NSAIDs, ARBs and ACEs use was added to the final model (Model 4). In model 4, all variables defined according to the literature that, a priori, would be potential confounding factors, regardless of the p-value, were kept. The significance level adopted was p < 0.05.

In the analysis of kidney function change, mixed linear regression models with random intercept and slope were used to assess longitudinal changes in GFR between baseline and wave 2. Such models are suitable for unbalanced and/or unevenly distributed data throughout time and data where the inter-subject variability is greater than the intra-subject one [38–40]. The fixed effects (β) and variance components (α) of the mixed linear models were estimated using restricted maximum likelihood methods.

In linear regression models with mixed effects, the exposure regression coefficients indicate the mean variation of the result at baseline and each moment (wave 2 in this paper). The interaction terms between a fixed-effect variable (PPI use and duration of use) and time determine whether that variable predicts longitudinal changes in the dependent variable over time. Therefore, we evaluated the interaction terms between age and the explanatory variables of interest, but only statistically significant terms (p < 0.05) were kept in the models.

We included the explanatory variables (PPI use and duration of use) and all covariates in the models as fixed effects, and age was modeled a random effect to index the time. All models included random effects on the age intercept and slope, allowing the individual’s initial value and longitudinal trajectory to vary with the population trajectory and average [38].

First, the GFR analysis was conducted using the explanatory variables of interest. We entered the covariates (sex, per-capita-household income, excessive alcohol consumption, smoking, obesity, cardiovascular disease, diabetes, hypertension, and NSAIDs, ARBs and ACEs use) into the models, step by step. We maintained in the final variables considered as confounding factors, according to the literature, regardless of the p-value. Finally, the interaction terms were added: PPI use x age and duration of PPI use x age. We presented the results only from the final models.

We conducted analyzes on Stata 14.0 (Stata Corporation, College Station, TX, USA).

## Results

The mean age of the participants without CKD was 51.4 (SD = 8.7) years and 57.4% were women. The PPI users were older, had lower per capita household income, were less smokers and more obese. Moreover, the prevalence of CVD, diabetes, hypertension, and use of NSAIDs, ARBs and ACEs was higher among PPI users (Table 1). Additionally, 7.6% (N = 1,005) reported regularly used of PPIs, with 1.2% (N = 161) using for up to six months and 6.4% (N = 844) above six months.

**Table 1:** Characteristics at baseline of the participants without chronic kidney disease according to use or not of proton pump inhibitors (PPI). ELSA-Brasil. (N = 13,301)

Characteristics	Does not use PPI (N = 12,296)	Use PPI (N = 1,005)	Total (N = 13,301)
	% or average (SD)ª	% or average (SD)ª	% or average (SD)ª
Age (years)	51.1 (8.7)	54.4 (9.0)	51.4 (8.7)
Sex			
Female	54.7	57.4	54.9
Male	45.3	42.6	45.1
Per capita household income			
R$ < 691.5	15.4	20.9	15.8
R$ 691.5	22.6	26.9	22.9
R$ 1037.25	21.1	19.9	21
R$ 1763.62	20.4	17.4	20.2
R$ 2628.17	20.5	14.9	20.1
Excessive Alcohol Consumption	7.4	6.8	7.4
Smokers			
Never	58	55.7	57.8
Ex-smokers	29	34.2	29.4
Current Smokers	13	10.1	12.8
Obesity	21.9	26.3	22.2
Cardiovascular Disease	5.8	9.7	6.1
Diabetes Mellitus	8.7	11.1	8.9
Hypertension	32.5	45	33.4
Use NSAIDs	1.6	3.4	1.8
Use BRAs	7	13.8	7.5
Use ACEs	9.6	13.9	9.9

ª SD = Standard Deviation.

Compared to non-users, PPI users had a lower mean glomerular filtration rate at baseline and wave 2. In addition, the prevalence of eGFR < 60 ml/min/1.73 m2 was higher among PPI users in wave 2 (Table 2).

**Table 2:** Mean and Standard Deviation (SD) of glomerular filtration rate estimated (eGFR) and incident chronic kidney disease (CKD) (%) according to use or not of proton pump inhibitors (PPI). ELSA-Brasil. (N = 13,301)

Characteristics	Does not use PPI (N = 12,296) % or average (SD)	Use PPI (N = 1,005) % or average (SD)	Total (N = 13,301) % or average (SD)
eGFR (baseline)	87.2 (13.5)	84.9 (14.0)*	87 (13.6)
eGFR (Wave 2)	84 (13.7)	81.4 (13.9)*	83.8 (13.7)
Incident CKD (Wave 2)^a^	3.6	6.5*	3.8

a eGFR < 60 ml/min/1.73 m^2^

* p < 0.05 in the chi-square test or t test.

In univariate logistic regression analysis, individuals who used PPIs at baseline had a risk of CKD 1.85 times greater than non-users (95% CI 1.41–2.41, p < 0.001). After adjusting for several confounding factors, the association showed no difference in significance and strength of correlation (Model 2, Table 3), but after adjusting particularly for hypertension the correlation remained borderline (OR = 1.29, 95%CI 0.97–1.71, p = 0.079).

**Table 3:** Association between the use of proton pump inhibitors and incident chronic kidney disease. ELSA-Brasil. (N = 13,301)

Models	Total N = 13,301 (100%)	Incident CKD N = 509 (3.8%)	Reference	OR (95%CI)
Model 0 ^π^			1	1.85 (1.41–2.41)*
Model 1^†^			1	1.36 (1.03–1.80)*
Model 2^‡^			1	1.35 (1.02–1.79)*
Model 3^£^			1	1.31 (0.98–1.74)
Model 4^§^			1	1.29 (0.97–1.71)

^π^ Model 0: not adjusted.

^†^ Model 1: adjusted for age, sex and per capita household income.

^‡^ Model 2: adjusted by model 1 + excessive alcohol consumption, smoking and obesity.

^£^ Model 3: adjusted by model 2 + cardiovascular disease, diabetes and hypertension.

^§^ Model 4: adjusted by model 3 + use of NSAIDs, ARBs and ACEs.

* p < 0.05.

In the univariate analysis, PPI users over six months had 2.33 times the risk of incident CKD compared to non-users (95% CI 1.69–3.22, p = 0.001) (Table 3). After adjusting for several confounders, the association remained borderline (Model 4; Table 4).

**Table 4:** Association between time of proton pump inhibitor use and incident chronic kidney disease. ELSA-Brasil. (N = 13,004)

PPI use time	Number incident CKD events^ϋ^	Reference^¥^	Model 0 ^π^ HR (95%CI)	Model 1^†^ HR (95%CI)	Model 2^‡^ HR (95%CI)	Model 3^£^ HR (95%CI)	Model 4^§^ HR (95%CI)
Up to 6 months	5	1	0.85 (0.35–2.09)	0.75 (0.30–1.86)	0.77 (0.31–1.91)	0.74 (0.30–1.86)	0.74 (0.30–1.86)
Over 6 months	44	1	2.33 (1.69–3.22)*	1.48 (1.05–2.07)*	1.46 (1.04–2.04)*	1.42 (1.01–2.00)*	1.39 (0.99–1.96)

^ϋ^ Number of cumulative incident CKD events during 3.9-year follow-ups

^¥^ Reference: Not PPI users.

^π^ Model 0: not adjusted.

^†^Model 1: adjusted for age, sex and per capita household income.

^‡^Model 2: adjusted by model 1 + excessive alcohol consumption, smoking and obesity.

^£^Model 3: adjusted by model 2 + cardiovascular disease, diabetes and hypertension.

^§^Model 4: adjusted by model 3 + use of NSAIDs, ARBs and ACEs.

*p < 0.05.

In the analysis of change in glomerular filtration, the average age of participants was 51.8 years old (SD = 9.0), and 54.7% were women. PPI users were older, had lower per capita household income, were less smoking and more obese. The prevalence of CVD, diabetes, hypertension and of NSAIDs, ARBs and ACEs use was also higher among PPI users (Table 5). At baseline, 7.9% (N = 1,096) of participants used PPIs, with 1.2% (N = 173) using for up to six months and 6.7% (N = 923) above six months. The average interval between visits was 3.9 years (range: 1.7 to 6.0 years).

**Table 5:** Characteristics of the study population according to use or not of proton pump inhibitors. ELSA-Brasil. (N = 13,919)

Characteristics	Does not use PPI (N = 12,823)	Use PPI (N = 1,096)	Total (N = 13,919)
	% or average (SD)ª	% or average (SD)ª	% or average (SD)ª
Age (years)	51.6 (8.9)	55.3 (9.3)	51.8 (9,0)
Sex			
Female	54.5	56.8	54.6
Male	45.5	43.2	45.4
Per capita household income			
R$ < 691.5	15.7	22.2	16.2
R$ 691.5	22.6	25.8	22.8
R$ 1037.25	21	20.4	20.9
R$ 1763.62	20.2	16.9	20
R$ 2628.17	20.5	14.7	20.1
Excessive Alcohol Consumption	7.4	6.5	7.3
Smokers			
Never	57.8	55.1	57.6
Ex-smokers	29.4	35	29.8
Current Smokers	12.8	9.9	12.6
Obesity	22.1	26.9	22.5
Cardiovascular Disease	6.2	11.1	6.5
Diabetes Mellitus	9.1	12.5	9.4
Hypertension	33.7	47.5	34.8
Use NSAIDs	1.7	3.5	1.8
Use BRAs	7.5	15.2	8.1
Use ACEs	10.2	15.2	10.6

ª SD = Standard Deviation.

After adjusting for all covariates, the interaction PPI use x age was statistically significant at -0.165 (95%CI -0.246; -0.084), indicating that PPI users had a more pronounced decline in eGFR between visits than non-users (Table 6).

**Table 6:** Association of baseline proton pump inhibitor use and change in glomerular filtration rate estimated by mixed-effect linear regression. ELSA-Brasil (N = 13,919)

Variable	Estimated glomerular filtration rate (eGFR)
	β (95%CI)
Intercept	122.347 (120.887 ; 123.807)*
Use of PPI	8.748 (4.258 ; 13.238)*
Age†(years)	-0.749 (-0.774 ; -0.723)*
Use of PPI x Age†	-0.165 (-0.246 ; -0.084)*

* β; p < 0.05.

† Age (age at baseline + segment time) was modeled as a random effect to index time.

Final model adjusted for: age†, sex, per capita household income, excessive alcohol consumption, smoking, obesity, cardiovascular disease, diabetes, hypertension, NSAIDs, ARBs and ACEs use.

The interaction term duration of PPI use x age was statistically significant in the categories of duration of drug use up to six months − 0.196 (95%CI -0.370; -0.021) and over six months − 0.149 (95%CI -0.248; -0.049), indicating that participants who, at baseline, used PPIs for six months or more had a more pronounced decline in eGFR between the two visits compared to non-users (Table 7).

**Table 7:** Association of duration of proton pump inhibitor use and change in glomerular filtration rate estimated by mixed-effect linear regression. ELSA-Brasil (N = 13,606)

Variable	Estimated glomerular filtration rate (eGFR)
	β (95%CI)
Intercept	124.148 (122.764 ; 125.532)*
PPI use time up to 6 months	10.590 (1.814 ; 20.365)*
Over 6 months	8.297 (2.387 ; 14.206)*
Age† (years)	-0.754 (-0.777 ; -0.731)*
Time of use PPI ^b^ x Age†	
Up to 6 months	-0.196 (-0.370 ; -0.021)*
Over 6 months	-0.149 (-0.248 ; -0.049)*

b Reference: Non PPI users.

*β; p < 0.05.

† Age (age at baseline + segment time) was modeled as a random effect to index time.

Final model adjusted for: age†, sex, per capita household income, excessive alcohol consumption, smoking, obesity, cardiovascular disease, diabetes, hypertension, NSAIDs, ARBs and ACEs use.

**Fig. 2:**
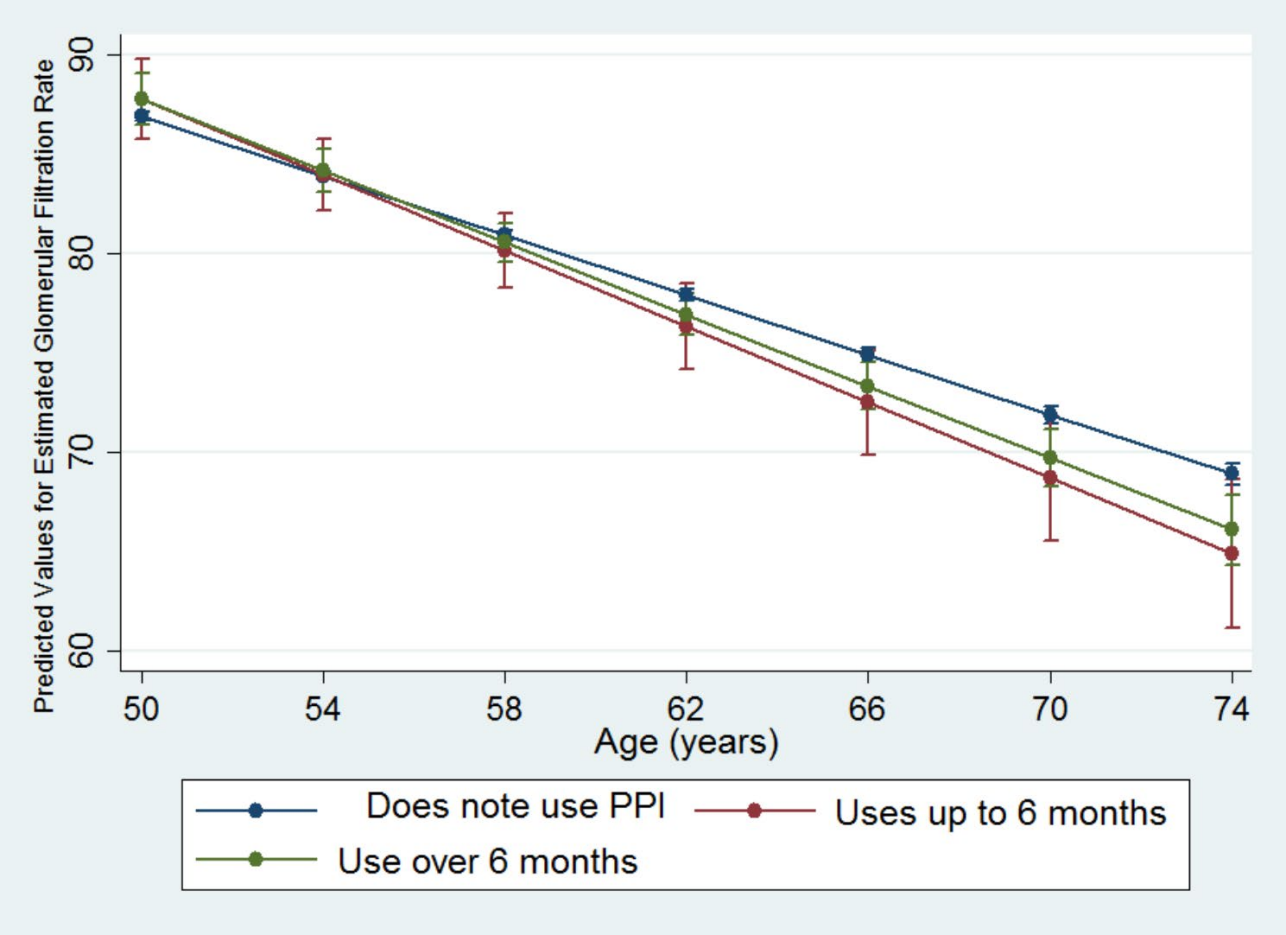
Longitudinal trajectories* of estimated glomerular filtration rate (eGFR) according to time of use of proton pump inhibitors (PPI). ELSA-Brasil (2008–2010 and 2012–2014). *As age was modeled as a random effect in the data analysis, this figure shows changes in estimated glomerular filtration rate over time (i.e., as individuals age). †Predicted numbers are dependent variable values based on estimated regression coefficients and a prediction of independent variable values after adjustments (age, sex, per capita household income, excessive alcohol consumption, obesity, cardiovascular disease, diabetes, hypertension, use of NSAIDs, BRAs, ACEs and interaction: time of PPI use × age)

We plotted an illustrative graphical representation of the estimated average for predicted eGFR levels concerning the explanatory variable time of PPI use (Fig. 2). Since age was modeled as a random effect in the analysis, the slopes in the figure indicate eGFR mean, stratified by exposure groups, as individuals aged during the follow up period.

## Discussion

Results from this study suggest a correlation between PPI use and renal function decline. Regular PPI use at baseline was associated with a more pronounced decline in eGFR between visits 1 and 2 in ELSA-Brasil and a higher incidence of CKD (borderline) after adjusting the confounders. The borderline result suggests a correlation since there was no change in the association strength after adjusting for several confounding factors (especially chronic diseases).

In a prospective cohort, Xie et al. evaluated 144,032 participants and after a median follow-up of 5 years that PPI users had a risk of reduced eGFR 1.22 (95%CI 1.16–1.28) times compared to PPI users of histamine H2 receptor antagonist (H2RA) [28]. In another prospective cohort, evaluating 173,321 PPI users and 20,270 H2RA users followed for over 5 years, Xie and colleagues found that PPI users had a risk of 1.32 (95%CI 1.28–1.37) times in the decrease in eGFR compared to H2RA users [29]. Klatte et al., in a retrospective cohort, evaluating 105,305 PPI users and 9,578 H2RA users after a median follow-up of 2.7 years (range 1.5 to 3.8), found a risk of 1.26 (95%CI 1.16–1.36) times for eGFR decline among PPI users [41].

Recent studies have suggested that PPI use is an independent risk factor for CKD development [27–29, 38, 41–44], but our study displayed a borderline association between PPI use and CKD incidence. Some differences between this study and others deserve to be highlighted. First, the follow-up time in our study (mean 3.9 years) was shorter than in the others (5 to 13 years) [29, 41–43]. Second, our study compared PPI users and non-users, unlike other studies that evaluated PPI users and H2RA users [41, 43]. Finally, the measurement of PPI exposure differs between studies.

We investigated time of use as a modifier of the effect of the association between PPI use and increased risk of incident CKD [29] and impaired renal function [28, 29, 44]. Rodriguez-Poncelas et al., evaluating 5,636 individuals, found an association between exposure to PPIs and the risk of incident CKD among PPI users from three to six months (HR = 1.42; 95%CI 1.11–1.80) and over six months (HR = 1.16; 95%CI 1.08–1.57) compared to non-users [36]. Xie et al. evaluated the association between PPI exposure time and the risk of renal outcomes among new PPI users (N = 173,321) and found that compared to individuals exposed for ≤ 30 days, there was a gradient between the duration of exposure and the risk of renal outcomes among those exposed for 31–90, 91–180, 181–360, and 361–720 days [29]. The association decreased with an exposure longer than 720 days, which is likely a reflection of a survival bias, an effect commonly referred to as “depletion of susceptibles” in pharmacoepidemiology, where individuals resistant to PPI effects on renal function remain in the cohort [29, 41, 45].

Our results suggest that association between drug use and loss of renal function may varies according to duration of PPI use. We found an association between PPI use over six months and incident CKD and PPI use up to six months and over six months and decline of renal function. These results corroborate the findings of recent studies on the association between PPI use and renal outcomes; and the change of association by the continuity of PPI use [29, 41]. There is no standardization in the literature for the categorization of the time of use of the PPI, which becomes a challenge for the comparison of our results.

Although the mechanisms that support the association between PPI use and CKD are not yet established, the relationship between PPI exposure and the risk of AKI and AIN is well established [16–18, 46], with AKI being associated with an increased risk of CKD [20]. And although the association between CKD and PPI exposure is postulated to be intervened by AKI [27, 42], a significant association between PPI use and CKD independent of AKI has been reported, which suggests that monitoring AKI and AIN in PPI users is not enough to protect against CKD [41].

Another possible mechanism is related to severe hypomagnesemia [22] that may be associated with the use of PPIs. Hypomagnesemia is associated with a more pronounced decline in eGFR in individuals with CKD, type II diabetes mellitus, progression to ESRD, and incidence of CKD [23–25]. Chronic PPI use can cause endothelial dysfunction leading to CKD through a variety of mechanisms, causing accelerated endothelial aging [47].

Our results corroborate other studies in the literature, showing an association between PPI use and decline in renal function. Nonetheless, we need to address some limitations. First, there may have been an error in classifying non-users of PPIs since any participant who used PPIs and was not using them only in the last two weeks (reference time of drug use) were classified as non-users. Thus, the PPI use prevalence may have been underestimated, underestimating the association. Second, the retention rate in the second wave was very high (94%), eligible individuals for this study who did not participate in wave 2 were older, with less education, higher prevalence of hypertension, diabetes and PPI use according to the analyzes performed (data not shown). Although the losses are small, these factors are associated with the decline in renal function, which may have contributed to overestimate the association found. In addition, it is important to note that, unlike clinical trials, observational study participants who use PPIs may have a higher risk of decline in eGFR for reasons unrelated to drug use. For example, our PPI users were more likely to be older, have lower per capita household income, be obese, and have a higher prevalence of cardiovascular disease, diabetes, and hypertension. Third, the individuals had their creatinine measured only once, which is inconsistent with the definition of CKD, because, although this does not meet the KDIGO definition, most large epidemiological studies have used a single eGFR definition for CKD [42]. Fourth, self-reported information about PPI use and comorbidities may have contributed to a recall bias, as some participants may have forgotten to mention a medication they were using or comorbidity. However, it is worth mentioning that the day before the visit, the participants received calls and were instructed to take the packages and prescriptions of all the medications they were using. Recognizing this potential bias, we adjusted for several confounding factors and the association remained. Besides, there is the possibility that variables not included in study and those not controlled could cause indication bias, as in all observational studies.

Strengths in our study that deserve to be highlighted are the studied population which included a large sample of relatively young individuals from a middle-income country, and the comprehensive data source that with laboratory information on GFR values, medication use and comorbidities. The ELSA-Brasil database represents an excellent tool for studying pharmacoepidemiology and pharmacovigilance because of the information collected on the participant’s medication use at each follow-up visit every three years on average [30]. This is particularly important for Brazil, which still has a very scarce and fragmented drug information system [48]. We also used a statistical model that considers the hierarchical structure of the data and allows the analysis of unbalanced or unevenly distributed longitudinal data.

## Conclusion

This study showed that PPI use and duration of its use of up to six months and above are associated with reduced eGFR. We also showed that PPI use for over six months is associated with an increase in the risk of developing CKD in a large sample of adult and elderly Brazilian people. Although observational studies do not have the best design to determine cause and effect, due to the large number of individuals currently using PPIs, healthcare professionals need to be cautious when prescribing, as well as monitoring the use of these drugs, due to the potential effects adverse.

## Data Availability

Data Sharing The datasets generated during analysed during the current study are available from the corresponding author on reasonable request.
